# Bandgap Engineering in the Configurational Space of Solid Solutions via Machine Learning: (Mg,Zn)O Case Study

**DOI:** 10.1021/acs.jpclett.1c01031

**Published:** 2021-05-25

**Authors:** Scott D. Midgley, Said Hamad, Keith T. Butler, Ricardo Grau-Crespo

**Affiliations:** † Department of Chemistry, University of Reading, Whiteknights, Reading RG6 6DX, United Kingdom; ‡ Department of Physical, Chemical and Natural Systems, Universidad Pablo de Olavide, Ctra.de Utrera km.1, 41013 Seville, Spain; § SciML, Scientific Computing Department, Rutherford Appleton Laboratory, Harwell OX11 0QX, United Kingdom

## Abstract

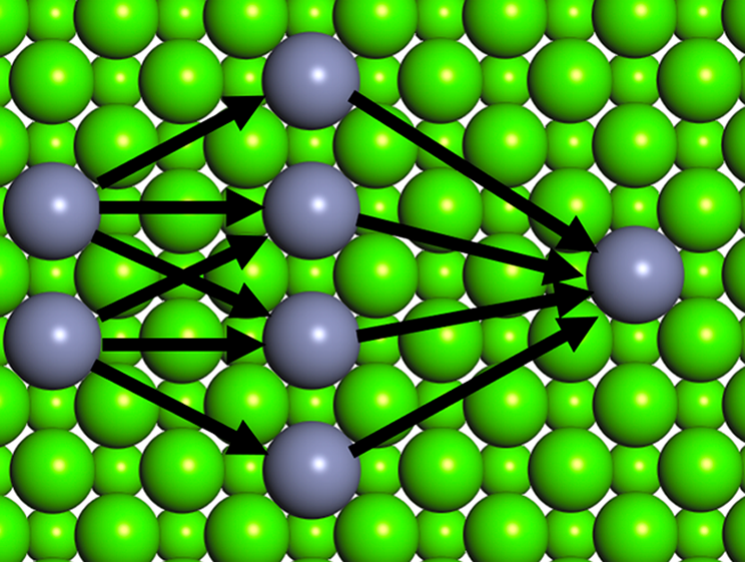

Computer simulations of alloys’ properties often require calculations in a large space of configurations in a supercell of the crystal structure. A common approach is to map density functional theory results into a simplified interaction model using so-called cluster expansions, which are linear on the cluster correlation functions. Alternative descriptors have not been sufficiently explored so far. We show here that a simple descriptor based on the Coulomb matrix eigenspectrum clearly outperforms the cluster expansion for both total energy and bandgap energy predictions in the configurational space of a MgO–ZnO solid solution, a prototypical oxide alloy for bandgap engineering. Bandgap predictions can be further improved by introducing non-linearity via gradient-boosted decision trees or neural networks based on the Coulomb matrix descriptor.

Density functional theory (DFT) is the most widely used electronic structure simulation technique in modern materials theory research. Despite its widespread use, DFT can incur a very high computational cost, making access to a high-performance computer a requisite for many applications, and prompting research into cheaper and more efficient ways to compute electronic properties of materials.

In recent years, machine learning (ML) has seen growing research interest in theoretical materials science because of its potential to reduce computational cost by several orders of magnitude compared with traditional DFT-only approaches.^1−4^ The development of atomic-level descriptors such as the Coulomb matrix has led to great progress in the accelerated prediction of molecular and material properties.^5,6^ The Coulomb matrix, defined as
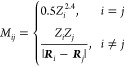
where *Z*_*i*_ and ***R***_*i*_ are the atomic numbers and positions of the atoms in the structure, was first used by Rupp and co-workers to show that a Gaussian regression method was able to accurately predict atomization energies in gas-phase molecules, significantly reducing the computational cost of a standard *ab initio* approach.^7^ Typically the matrix is flattened to vector form by using the sorted spectrum of eigenvalues, leading to a convenient vector shape for the descriptor (the Coulomb matrix eigenspectrum or CME), which is invariant to translation, rotations, and permutations of atom indices. The CME descriptor has been generalized to periodic systems and employed for the description of formation energies in solids.^8^

The investigation of the vast configurational space of solid solutions is another area where ML can accelerate predictions. The most established approach to calculate the energies (and sometimes other properties) of solid solution configurations is to create a so-called cluster expansion, where the energy is represented as a linear expansion of cluster correlation functions (CCFs) of increasing order, i.e., points, pairs, trios, quartets, etc.^9^ Cluster expansions have been hugely successful in the theoretical understanding of alloys, but they also have limitations, for example, related to relaxation effects and numerical errors.^10,11^ Rosenbrock et al. have recently proposed ML potentials as an alternative to cluster expansions for the investigation of alloy phase diagrams.^12^ Natarajan and van der Ven employed ML tools including neural networks to generalize the cluster expansion approach by relaxing the condition of linearity on the CCFs.^13^ An alternative approach, which we follow in this work, is to use a different descriptor altogether, one that is not constrained by the locality of the CCFs, like the CME mentioned above. This is especially worth exploring for the prediction of non-additive properties, such as bandgaps, where the cluster expansion might not perform as well as for energies.

Solid solutions offer the possibility of band structure engineering for many applications. Mg_1–*x*_Zn_*x*_O solid solutions, chosen here as a case study, constitute an important family of wide-gap semiconductors with tunable bandgaps from 3.3 to 7.8 eV.^14,15^ Thin films made of these solid solutions are of interest in the field of ultraviolet optoelectronic devices.^16−18^ Precise bandgap engineering is therefore needed, which can be achieved to a great extent via compositional optimization. We are interested here in the possibility of optimizing the bandgap in the configurational space (at fixed composition) rather than in the compositional space, since it is known that modern crystal-growth techniques, like molecular beam epitaxy, can produce targeted crystal structures, often in defiance of equilibrium thermodynamics. Previous DFT calculations performed in alloy models in a small 16-atom cell have already suggested the existence of large bandgap fluctuations due to differences in the local arrangement of Mg and Zn atoms.^19^ However, expanding these DFT-based studies to larger supercells to properly explore the configuration space would have a prohibitively large computational cost.

We present here an investigation of different computational approaches to map the bandgaps of alloy configurations into a simple model that allows fast prediction and screening across a large configurational space. We use the 3:1 MgO–ZnO rocksalt solid solution as a case study, both because it is a well-known system with important applications and because it does not pose extra challenges to DFT like partially filled *d* orbitals or spin polarization. We will compare the performance of CCF vs CME descriptors, as well as linear vs non-linear regression models, in the hope of discovering new routes for more accurate bandgap engineering in solid solutions.

The MgO and ZnO end members have cubic and hexagonal crystal structures, respectively.^20,21^ A 64-atom cubic supercell with composition Zn_8_Mg_24_O_32_ was chosen as a case study for the assessment of ML methods for the prediction of mixing energy (*E*_mix_) and band gap energy (*E*_gap_) in the solid solution. This composition and cell size give 8043 symmetrically inequivalent cation configurations, with configurations considered equivalent if they are related by a symmetry operator of the parent structure.^22^ We used the Supercell code to generate the inequivalent configurations.^23^ This number of configurations is both large enough to yield statistically meaningful data-driven conclusions and small enough to permit a full DFT treatment for training and validation of the ML models.

Symmetrically inequivalent configurations were subject to full geometry and cell vector optimization using DFT simulations with periodic boundary conditions, as implemented in the VASP code.^24^ The generalized gradient approximation (GGA) was used for the exchange-correlation term, with the functional by Perdew, Burke, and Ernzerhof (PBE).^25^ The projector augmented wave (PAW) method was used to describe the interactions between atomic cores and valence electrons.^26,27^ A plane wave kinetic energy cutoff of 520 eV was used, which is 30% above the recommended value for the set of PAW potentials used, to minimize Pulay stress errors. The end members are modeled with high accuracy with this type of calculations, as we can see by the good agreement between DFT-optimized cell parameters and experimental values in Table 1.

**Table 1:** Relaxed Cell Parameters and Bandgaps of the Solid Solution End Members (MgO and ZnO) from DFT Calculations, in Comparison with Experimental Values

	MgO		ZnO	
crystal system (space group)	cubic (*Fm*3*m*)		hexagonal (*P*6_3_*mc*)	
	calc	exp	calc	exp
*a*/Å	PBE: 4.24	4.22	PBE: 3.24	3.25
*c*/Å	–	–	PBE: 5.18	5.21
*E*_gap_/eV	PBE: 4.5		PBE: 1.4	
	HSE: 6.2^a^	7.8^b^	HSE: 2.6^a^	3.3^c^

a Calculated using the HSE functional at PBE geometry.

b Ref (14).

c Ref (15).

It is well known that GGA-PBE gives a poor description of bandgaps, generally underestimating the experimental values. In order to find out how to correct the PBE values, a small subset of 20 configurations across the full range of bandgaps was chosen for more accurate calculations using the screened hybrid functional by Heyd, Scuseria, and Ernzerhof (HSE), which incorporates 25% Hartree–Fock exchange energy and is much better than GGA at predicting bandgaps.^28^ We demonstrate that for the ZnO/MgO alloy studied here, the PBE bandgaps may be easily corrected via a simple linear transformation to reproduce the HSE bandgaps. The linear relation between the bandgap values calculated with PBE and with HSE can be seen in Figure S1a in the Supporting Information (SI). This strong linear correlation between PBE and HSE bandgaps is not general, and in systems including transition-metal or rare-earth elements, for example, we would expect much weaker correlations. For such systems, the non-linear relationship between PBE and HSE bandgaps can be established using a machine-learned transformation.^29^ However, in our case the simple linear relationship will allow us to use PBE band gaps for training the bandgap predicting models, instead of the more expensive but more accurate HSE values. It can also be seen from Table 1 that, while giving better predictions than PBE, HSE still underestimates the experimental bandgaps for pure MgO and ZnO, in both cases by ∼20%. So it is reasonable to expect a similar underestimation by HSE of the solid solution bandgaps.

We used ML methods to learn from DFT-derived *E*_mix_ and *E*_gap_ values for a subset of configurations and to predict the values for the rest of the configurations. This procedure permits a significant reduction of the computational cost, brought about by a reduction in the number of DFT calculations required to obtain accurate *E*_mix_ and *E*_gap_ values for the entire configurational space. As descriptors of the alloy configurations, we used either the full vector of cluster correlation functions (CCFs) or the Coulomb matrix eigenspectrum (CME). The CCF vectors have 90 components, corresponding to all the symmetrically distinct clusters up to four-body terms, as calculated using the CELL code.^30^ More information about the CCF descriptor employed in this work is given in the SI. The 64-component CME vectors were generated using the Python 3 packages Matminer and Pymatgen.^31,32^

Linear regression (LR) and gradient-boosted decision tree (GBDT) methods were performed using Python 3 Scikit-Learn packages.^33^ For LR models, we added weak *LASSO* regularization to obtain physically meaningful parameters.^34^ Deep-learning neural networks of the feedforward multilayer perceptron (MLP) architecture were written using the Keras^35^ package, which is built on the TensorFlow^30^ platform. MLP models were subject to extensive architecture testing, though only two architectures, which we will refer to as *shallow* and *deep*, are discussed forthwith. The *shallow* architecture is a three-layer feedforward perceptron with 64-32-1 nodes per layer, whereas the *deep* architecture is a five-layer feedforward perceptron with 256-128-64-32-1 nodes per layer. Data was split into sets based on a percentage of the 8043 datapoints available: training (fractions between 10% and 80% were tried), validation (10%), and testing (10%). This ensured that ML vs DFT energy plots involved data that had not been used for either training or validation, and that the testing dataset size stayed constant when varying the training dataset size. More details about the ML algorithms can be found in the SI.

We briefly discuss the DFT results first, before moving into the regression models. Figure 1 reports the mixing energies plotted against the bandgaps as obtained by DFT calculations for the whole dataset of 8043 configurations. The wide range of bandgaps (∼1 eV difference between the minimum and maximum PBE values, which can be estimated to correspond to a range width of ∼1.5 eV in the experimental scale), together with the small stability difference between configurations (less than 0.3 eV per supercell, which is less than 0.01 eV per formula unit), confirms that this would be a suitable system for *configurational* bandgap optimization, at fixed composition. There is some weak but clear correlation between *E*_mix_ and *E*_gap_, suggesting that thermodynamics might oppose the arrangement of cation distributions in the ways that lead to maximum bandgaps. However, given the small energy differences, we would not expect thermodynamics to prevent the experimental realization of these wide-gap configurations.

**Figure 1:**
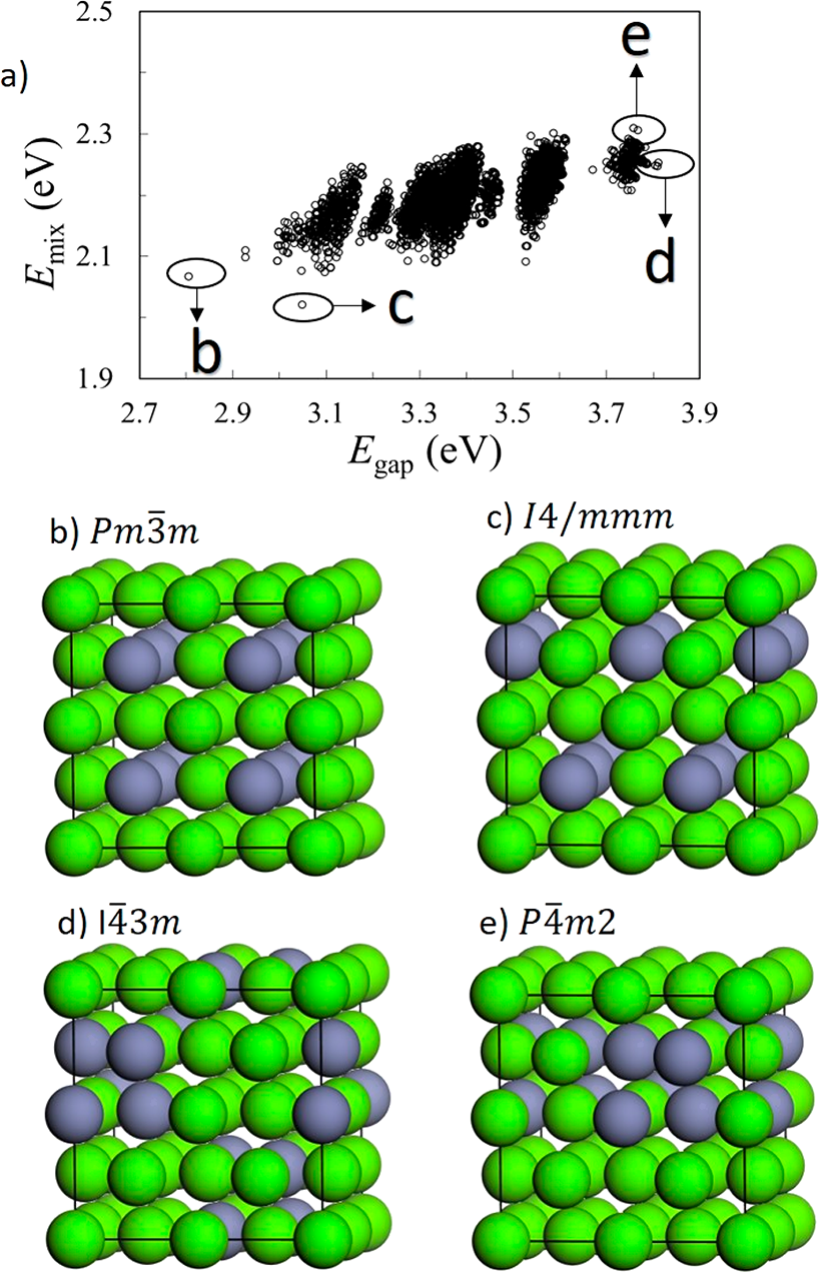
(a) DFT data (mixing energy vs band gap energy) for all 8043 symmetrically different Zn_8_Mg_24_O_32_ configurations. Structures of the configurations with (b) minimum *E*_gap_, (c) minimum *E*_mix_, (d) maximum *E*_gap_, and (e) maximum *E*_mix_. Green and gray balls represent Mg and Zn atoms, respectively (O atoms are omitted for clarity).

The geometries of the configurations with minimum and maximum values of *E*_mix_ and *E*_gap_ are also shown in Figure 1. The configuration with the lowest bandgap (Figure 1b) has the same distribution of ions as the ordered fcc alloy Cu_3_Au, i.e., has the structure with Strukturbericht designation L1_2_ and space group *Pm*3*m*. This configuration is characterized by −Zn–O–Zn–O– one-dimensional chains along the three equivalent [100], [010], and [001] directions of the crystal structure. Since ZnO has a much lower bandgap than MgO, it is not surprising that the presence of periodic ZnO-only chains tends to lower the bandgaps. The configuration with the lowest mixing energy, i.e., the configurational ground state for the composition Mg_3/4_Zn_1/4_O, is the one with Strukturbericht designation D0_22_ and space group *I*4/*mmm*, as in the ordered alloy Al_3_Ti, which agrees with the conclusion from the previous theoretical study by Sanati et al.^36^ This configuration also has −Zn–O–Zn–O– one-dimensional chains along two of the crystal axes, but the cations alternate in the third direction, forming −Mg–O–Zn–O– chains (Figure 1c). The configurations with the maximum values of *E*_gap_ (Figure 1d) and *E*_mix_ (Figure 1e) both have all Zn dopants forming alternating −Mg–O–Zn–O– chains, with no pure −Zn–O–Zn–O– chains along the crystal axes. However, in the most unstable configuration (maximum *E*_mix_), with space group *P*4*m*2, these chains aggregate within two neighboring layers (the cation size disparity between Zn and Mg is likely to cause high crystal strain when concentrated at one side of the cell, which explains the high mixing energy), whereas in the former the distribution of the chains forms a more homogeneous, checkered-like pattern with space group *I*43*m*.

The main purpose of this work is not, however, to identify configurations with extremal properties, but to devise fast and accurate methods to calculate the properties of any alloy configuration. Figure 2a shows the plot of predicted vs true data for the test set, using models based on the CCF (i.e., the cluster expansions), when 80% of the data was used for training. The correlation between the cluster expansions and the mixing energies obtained directly from DFT is rather poor. This is somewhat surprising, since cluster expansions generally perform well at describing energy differences between alloy configurations. For MgO–ZnO solid solutions, Yin et al. have previously presented a cluster expansion for the formation energies, which fitted well their DFT energies, with one-point and two-point clusters found to be dominant in the expansion.^37^ However, in that case the authors were examining configurations across a range of compositions, and therefore the one-point correlation functions were the dominant term, thus improving the correlation between predicted and target energies. In our case, we are working at a fixed composition (so we leave the one-point cluster correlation functions out of the regression) and the range of energies is very narrow, which is more challenging for the cluster expansion. So even when the mean absolute error for our cluster expansion is small (0.02 eV per supercell, which is less than 1 meV per formula unit), the correlation between the predicted and target data is still weak (*R*^2^ = 0.39).

**Figure 2:**
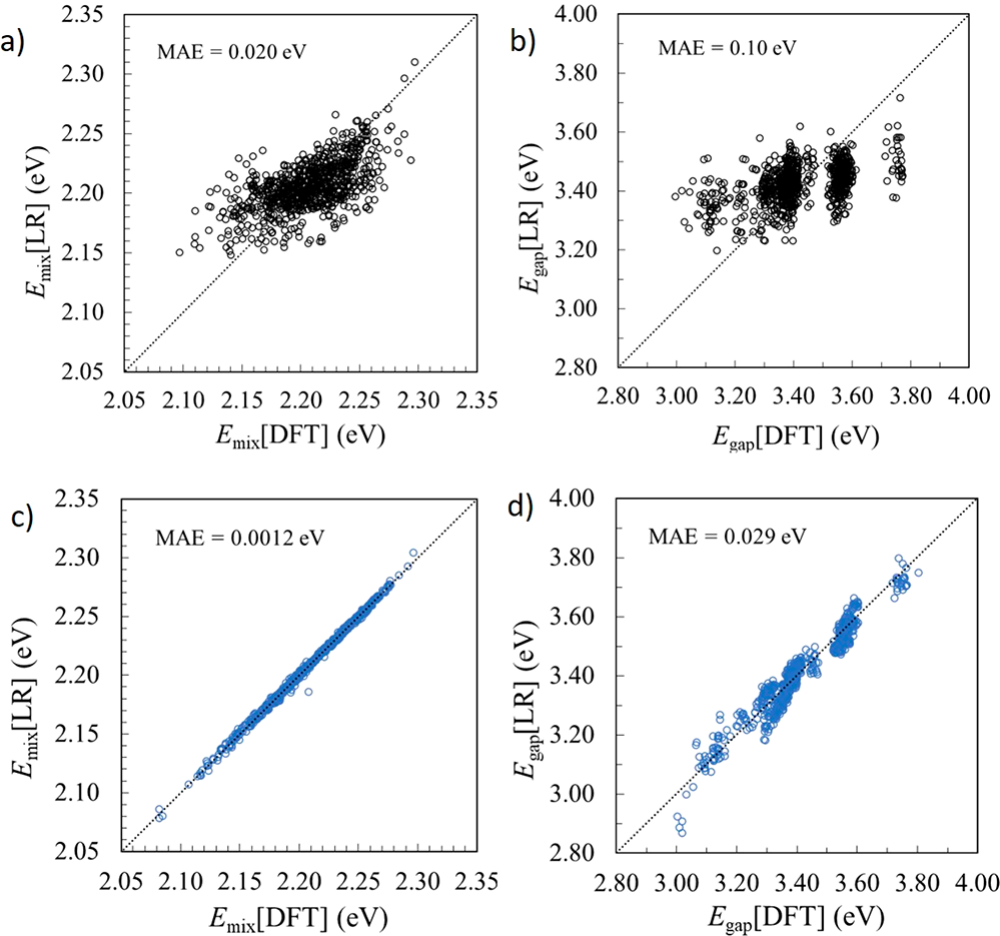
Performance of linear regression models, trained on 80% of the data, when used for the test set using (a) cluster correlation function (CCF) descriptor for *E*_mix_, (b) CCF descriptor for *E*_gap_, (c) Coulomb matrix eigenspectrum (CME) descriptor for *E*_mix_, and (d) CME descriptor for *E*_gap_.

The plot of predicted vs true data for the cluster expansion of the bandgaps, also based on training with 80% of the data, is shown in Figure 2b. In this case the correlation is even poorer (*R*^2^ = 0.22), which is not surprising, given that bandgaps are not additive and depend on the long-range pattern in the distribution of ions in the solid, which is not necessarily well captured by the local cluster functions. But even when cluster expansions of bandgaps are not as well established or justified as the cluster expansions of energies,^38^ the method has been widely used for bandgaps,^39,40^ and no reliable alternatives have been developed. Relaxing the linearity condition on the CCFs (as done recently in a different context in ref (13)) did not significantly improve the performance of the descriptor: a MLP model trained using the CCF descriptor yielded equally poor correlations (see SI).

In contrast, using the CME as a descriptor leads to excellent correlation between predicted and target data, as shown in Figure 2c,d. The prediction for *E*_mix_ is particularly outstanding, with a mean absolution error of ∼1 meV per supercell on the test set. Even the bandgap prediction is quite good, although with some more dispersion. The observation that the CME descriptor performs better than the CCFs, which are traditionally used for cluster expansions, is very interesting, since cluster expansions have been the preferred theoretical tool for the investigation of the configurational space of alloys for several decades. Using widely available tools, generating the CME is just as easy and computationally cheap as generating the CCFs, and we demonstrate here that it can lead to more accurate predictions. Of course, the advantage of a model based on the CCFs is that, once the cluster expansion is generated, it can be used to explore the configuration energies in supercells larger than those used in the fitting. This is useful to compute thermodynamic properties with converged cell sizes. However, this advantage does not translate trivially to the prediction of bandgaps or other non-additive quantities. In these cases, as we are constrained to make predictions within the same supercell size from where configurations are sampled for training, the CME descriptor might be a more accurate and equally cheap choice.

Finally, we consider whether non-linear regression models can further improve the CME-based description of the bandgaps, based on the CME descriptor. Figure 3a,b shows the bandgap prediction made by the *deep* MLP model (the *shallow* MLP results are reported in Figure S3 of the SI). Clearly, the MLP improves the prediction with respect to the linear regression model. Even when only 10% of the data is used for training, the predicted bandgaps are much better (with roughly half of the MAE) than those predicted with the linear regression model using 80% of the data for training. Furthermore, MLP models show significant improvement when increasing the dataset size, whereas the linear regression model does not seem to benefit from the use of additional training data. A comparison between the MLP methods in terms of performance for the *shallow* and *deep* architectures is reported in Table S1 of the SI. The *deep* MLP is deeper and wider than the *shallow* MLP and provides slightly improved performance because the increased complexity of this MLP may capture more non-linearities in the CME–*E*_gap_ relationship. There is a slightly increased risk of overfitting when using a more complex MLP, though we found no evidence of this during training.

**Figure 3:**
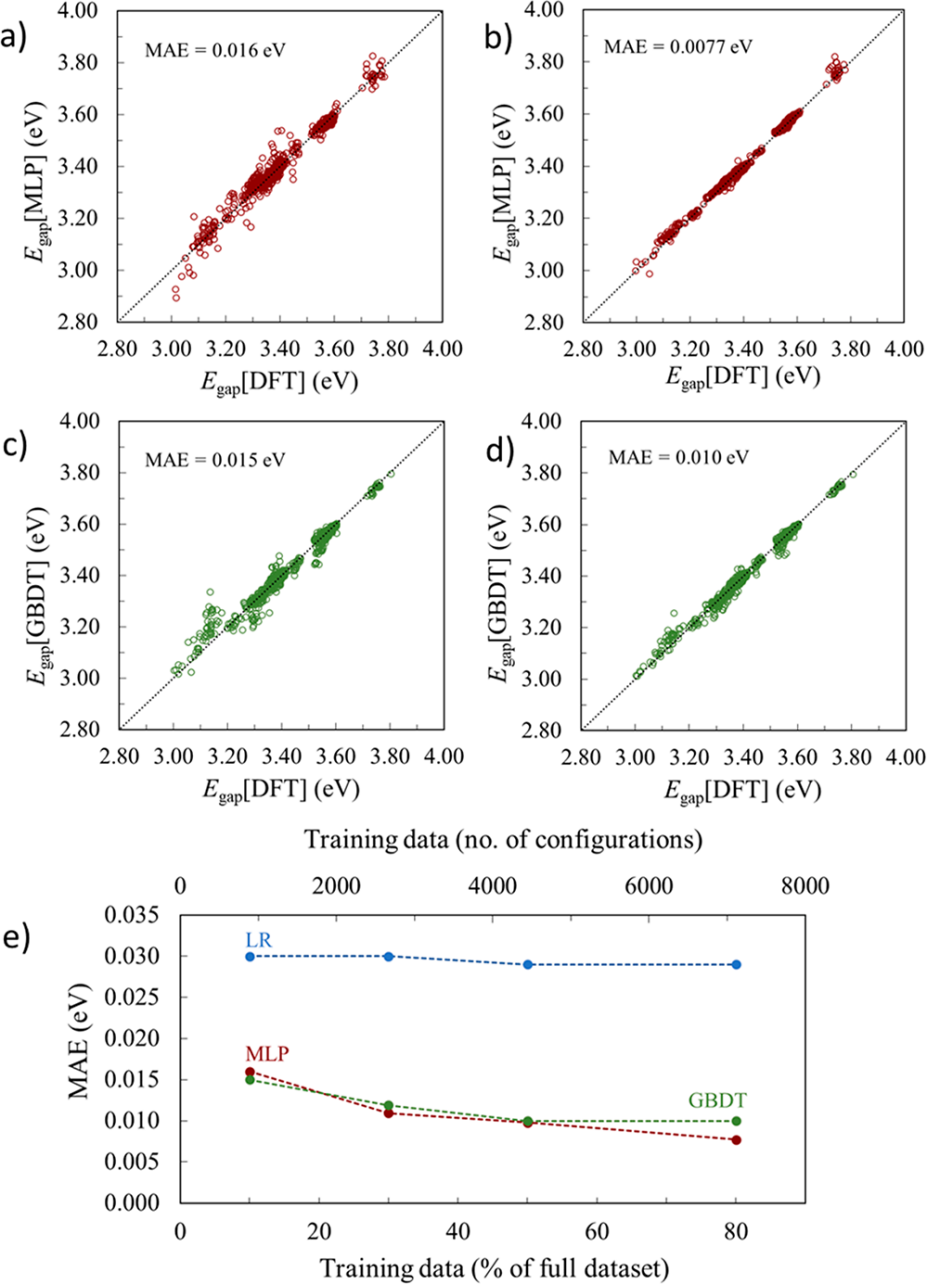
CME machine learning models for *E*_gap_: (a) *deep* MLP-10%, (b) *deep* MLP-80%, (c) GBDT-10%, (d) GBDT-80%, and (e) MAE vs % training data (of 8043 total configurations).

GBDT models (Figure 3c,d), trained using optimized hyperparameters reported in the SI, also proved to be very effective in predicting bandgaps, especially for the small- to medium-sized training sets. The performance of the GBDT model saturates after a certain size of training set between 50% and 80% of the data used here, meaning that it is unlikely to benefit as much as MLP from increasing the training dataset size. However, given that the associated mean absolute errors are similar to those of the MLP models, GBDT models constitute an attractive alternative, since the computational cost of training these models is smaller than for the neural networks. A full performance comparison for the three ML methods is given in the SI, Table S2.

In conclusion, we have shown that Coulomb matrix eigenspectrum descriptors outperform the cluster correlation functions typically used for cluster expansions in the prediction of both properties for a MgO–ZnO solid solution. Cluster expansions are more justified for configurational thermodynamics, because energy expansions are trivially extrapolated to the very large supercells required for accurate statistical mechanics. However, for the screening of bandgaps in the configurational space, cluster expansions are not ideal, not only because of the non-additive character of bandgaps which limits the extrapolation to larger supercells but also because the cluster expansions might not capture well the bandgap variations in the first place, as we have shown in this study. We suggest that, for this problem, a better approach is to sample the configurational space in an affordable supercell, perform DFT calculations, and then use modern machine learning tools, based on Coulomb matrix eigenspectrum descriptors and linear or non-linear regression models (depending on the size of the available datasets). Given the wide availability and low computational cost of these machine learning tools, we believe that this approach will become the new standard for the prediction of electronic properties in the configurational space of semiconducting alloys.

## Supporting Information Available

Bandgap correction using the screened hybrid functional HSE; calculation of the cluster correlation functions; performance of MLP neural network using CCF descriptor; comparison of shallow vs deep MLP neural networks using CME descriptor; further details about the machine learning algorithms; metrics summary; and information about codes and data (PDF)

## Notes

Associated codes and data are available online: https://doi.org/10.5281/zenodo.4736810 (data) and https://github.com/scott-midgley/Machine-Learning-for-Solid-Solutions (codes).

## Abbreviations

DFT: density functional theory
ML: machine learning
CME: Coulomb matrix eigenspectrum
CCF: cluster correlation function
GGA: generalized gradient approximation
PBE: Perdew, Burke, and Ernzerhof
PAW: projector augmented wave
HSE: Heyd, Scuseria, and Ernzerhof
LR: linear regression
GBDT: gradient-boosted decision tree
MLP: multilayer perceptron
MAE: mean absolute error
