# Supplementary material for: Bandgap Engineering in the Configurational Space of Solid Solutions via Machine Learning: (Mg,Zn)O Case Study
Source: J Phys Chem Lett. 2021 May 25;12(21):5163–8. doi: 10.1021/acs.jpclett.1c01031 (PMC8279729; doi:10.1021/acs.jpclett.1c01031)
Supplement: Supplementary file 1 — jz1c01031_si_001.pdf [file jz1c01031_si_001.pdf]

Bandgap Engineering in the Configurational Space of Solid Solutions via Machine Learning: (Mg,Zn)O Case Study

Scott D. Midgley,[†] Said Hamad,[‡] Keith T. Butler,[§*] Ricardo Grau-Crespo[†*]

[†] Department of Chemistry, University of Reading, Whiteknights, Reading RG6 6DX, United Kingdom. *E-mail: r.grau-crespo@reading.ac.uk

[‡] Department of Physical, Chemical and Natural Systems, Universidad Pablo de Olavide, Ctra.de Utrera km.1, 41013 Seville, Spain.

[§] SciML, Scientific Computer Division, Rutherford Appleton Laboratory, Harwell OX11 0QX, United Kingdom. *E-mail: keith.butler@stfc.ac.uk

Supplementary Information

Contents

1. Bandgap correction using the screened hybrid functional HSE 2
2. Calculation of the cluster correlation functions 3
3. Performance of MLP neural network using CCF descriptor 5
4. Comparison of *shallow* vs *deep* MLP neural networks using CME descriptor 6
5. Further details about the machine learning algorithms 7
6. Metrics summary 8
7. Information about codes and data 9

References 10

1. Bandgap correction using the screened hybrid functional HSE

The PBE functional generally gives poor values of bandgap. The bandgaps obtained with HSE calculations tend to be in better agreement with experiment, but at a much higher computational cost. Interestingly, we found that PBE and HSE derived values of bandgaps are highly correlated for this system. In order to obtain a linear relation that would allow us to calculate near-HSE-quality values of bandgap from PBE bandgaps, we first chose five random configurations from each of the four bandgap energy quartiles (Figure 1a), thus ensuring the representation of the full E_{gap} range. The correlation between HSE vs PBE bandgap energies is shown in Figure S1 (a).

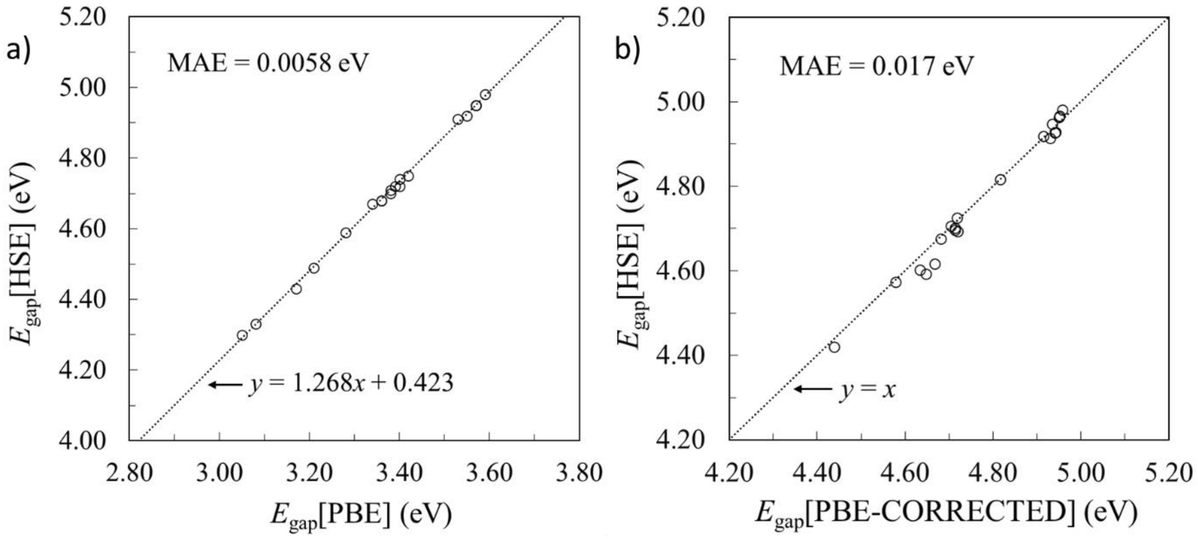

Figure S1. a) Linear regression of HSE *vs.* PBE bandgaps using 20 randomly selected configurations; b) test of the linear regression using a separate set of 20 random configurations not including in the fitting.

The linear fit that best describes the correlation between E_{gap}[HSE] and E_{gap}[PBE] is:

$$E_{\mathrm{gap}}[\mathrm{HSE}] = 1.268\, E_{\mathrm{gap}}[\mathrm{PBE}] \;+\; 0.423\ \mathrm{eV} \tag{S1}$$

Equation S1 provides us with a way to correct the PBE values and get bandgaps as similar as possible to HSE ones, at a fraction of the cost.

To test the validity of this approach, we applied the linear correction to a further 20 randomly selected configurations (across each of the four quartiles). These PBE-corrected E_{gap} values were plotted against the corresponding HSE bandgaps, as shown in Figure S1b. It is clear that the linear transformation model is effective for correcting values on configurations for which it was not trained.

2. Calculation of the cluster correlation functions

The simulation cell that we have employed in all our calculations consists of a 2x2x2 repetition of the parent MgO unit cell. The space group of the parent structure is 225. Since the cubic unit cell of MgO has a lattice parameter of 4.248 Å, the 2x2x2 supercell used in our calculations has a length of 8.496 Å. There are 32 cation sites in this supercell, of which 24 are occupied by Mg cations and 8 by Zn cations. In order to calculate efficiently the energies of the different $(Mg_{24}Zn_8)O_{32}$ configurations we make use of cluster expansion (CE) theory.[1] Within this approach, the energy of a particular configuration, s, of the solid solution, can be calculated as a linear expansion:

$$E_{\mathrm{s}} = \sum_{\alpha}^{N\mathrm{c}} m_\alpha J_\alpha X_{s\alpha}$$

over the so-called cluster correlation functions (CCFs) $X_{s\alpha}$, where the sum extends over all clusters α of exchangeable sites (e.g. points, pairs, trios, quartets, etc.), and J_α are the effective cluster interactions (ECIs), $N\mathrm{c}$ is the number of symmetrically distinct clusters, m_α are the multiplicities (number of symmetrically equivalent clusters of type α). The CCFs $X_{s\alpha}$ can be defined as the average of the product of a basis function ϕ of occupation variables σ_{si} (-1 and 1 for each element in a binary alloy) over all the clusters of type α:

$$X_{s\alpha} = \frac{1}{m_\alpha} \sum_{\beta \equiv \alpha} \prod_{i \in \beta} \phi(\sigma_{si})$$

There is some flexibility in the choice of basis functions. We used two types of basis functions:[1] i) a non-orthonormal, binary basis, $\phi(\sigma_{si}) = \frac{1+\sigma_{si}}{2}$ which takes values 0 and 1 for Mg and Zn occupancy, respectively; and ii) an orthonormal basis function using discrete Chebyshev polynomials. The results obtained when using both types of cluster basis are very similar, so in the manuscript we only report the results using the orthonormal basis function.

We have employed the CELL tool[2] to obtain the CCF vectors $\mathbf{X}_{\mathrm{s}} = \{X_{s\alpha}\}$ for each of the 8043 symmetrically different configurations of the supercell with composition $Mg_{24}Zn_8O_{32}$. There are 92 symmetrically distinct clusters up to fourth order, namely 1 empty cluster, 1 one-point cluster, 5 two-point clusters, 14 three-point clusters and 71 four-point clusters. Figure S2 shows some of these clusters. Because we are only comparing configurations with the same composition, the CCFs for the empty cluster and for the one-point clusters are constant for all configurations. The vectors $\mathbf{X}_{\mathrm{s}}$ formed from the remaining 90 correlation functions are then used as configurational descriptors for the linear regression (i.e. for finding the ECIs) or non-linear analysis using different techniques, as described in the text.

a)

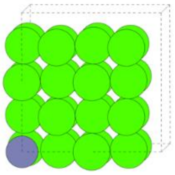

b)

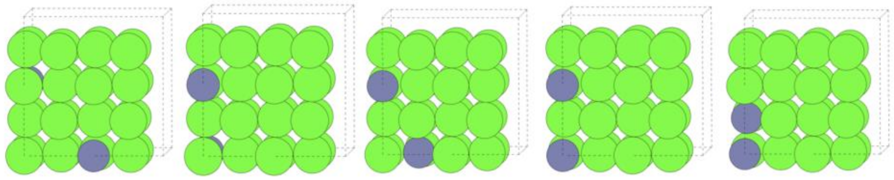

c)

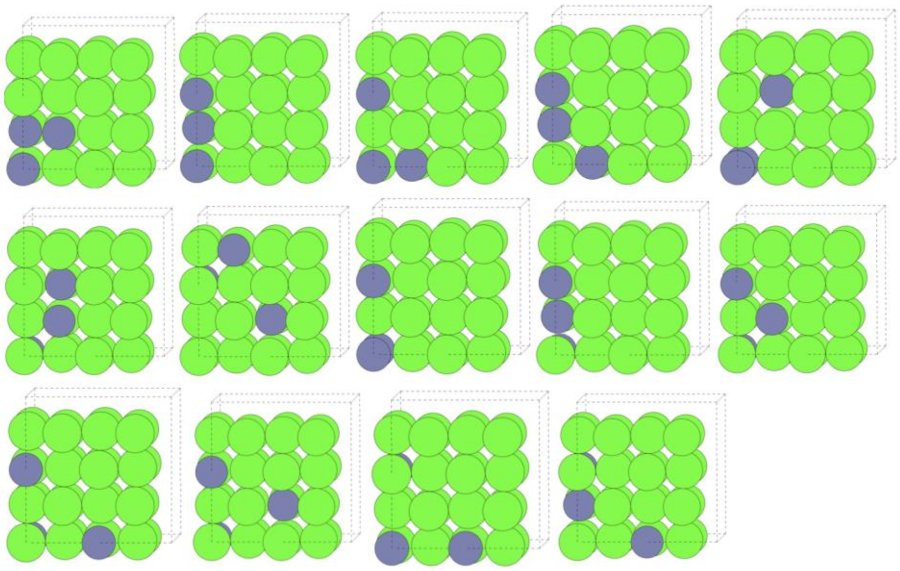

d)

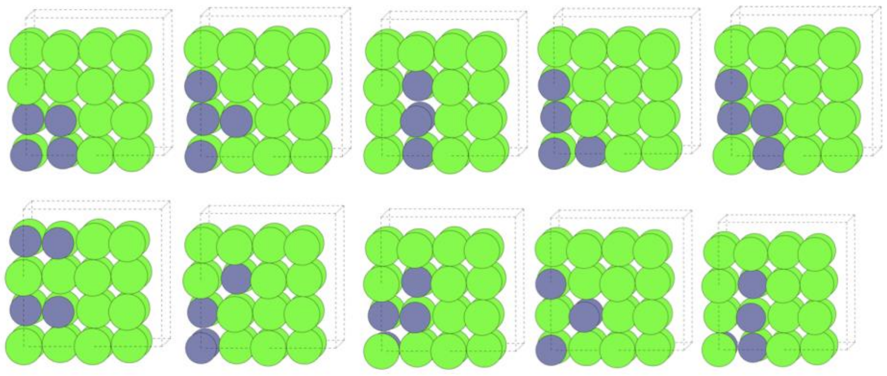

Figure S2. Atomistic view of a) the only 1-point cluster, b) the five 2-point clusters, c) the fourteen 3-point clusters, and d) ten of the 71 4-point clusters. Oxygen atoms are not shown, for simplicity reasons. The sites forming the clusters are shown in blue, and the rest of the cation sites in green.

3. Performance of MLP neural network using CCF descriptor

We found that relaxing the linearity condition on the CCFs did not significantly improve the performance of the descriptor for mixing energies or bandgaps. Training the *deep* MLP model using the CCF descriptor yielded equally poor correlations as shown in Figure S3.

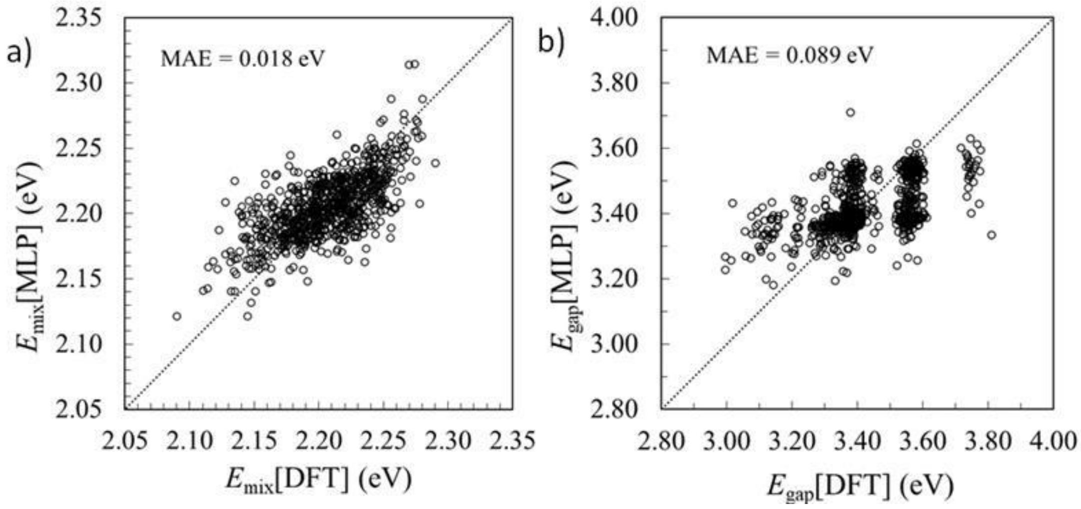

Figure S3. *Deep* multilayer perceptron E_{mix} and E_{gap} predictions using the CCF descriptor and 80% of the configurations for training.

4. Comparison of *shallow* vs *deep* MLP neural networks using CME descriptor

We compared the performance of the *deep* vs *shallow* MLP models for predicting bandgaps. Though the *deep* MLP outperformed the *shallow* MLP in terms of MAE, we report the results of the *shallow* MLP here for comparison. *Shallow* MLP architectures may have the advantage of avoiding overfitting in certain use-cases, although there was no evidence of overfitting encountered in the present work. The performance of the *shallow* MLP increased as the training dataset size increased, as is the case of the *deep* MLP (reported in the main text).

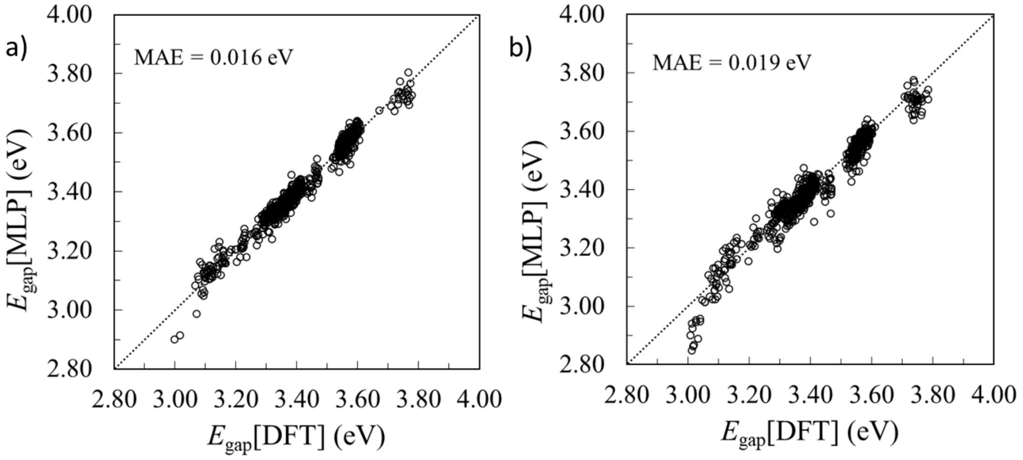

Figure S4. *Shallow* multilayer perceptron E_{gap} prediction based on the CME descriptor for a) 80% of the configurations used in training; b) 10% of the configurations used in training.

Table S1 reports a comparison of the *shallow* and *deep* MLPs in terms of performance. Although the *shallow* MLP performs very well and incurs in slightly shorter training times, the *deep* MLP is superior when using the same amount of data for training.

Table S1. Summary of *shallow* and *deep* MLP neural network performances when using the CME descriptor to predict bandgaps.

	Shallow network			*Deep* network		
**% Training Data**	**Epochs**	**MAE**	**Time* (min)**	**Epochs**	**MAE**	**Time* (min)**
**10**	9060	0.019	6	6484	0.016	8
**30**	5732	0.018	8	3057	0.011	8
**50**	3022	0.017	6	3385	0.0098	13
**80**	2491	0.016	7	3240	0.0077	16

*Training time on Intel i7 Coffee Lake processor (@4.2 GHz).

** For reference, the computing time required for training with all GBDT methods required less than five minutes for training, while LR requires less than ten seconds in each case.

5. Further details about the machine learning algorithms

GBDT was subject to hyperparameter optimization. The following settings were employed in the process (the corresponding name in the class *sklearn.ensemble.GradientBoostingRegressor* is shown in parenthesis):

- Learning rate (*learning_rate*) = 0.01
- Maximum depth of the individual regression estimators (*max_depth*) = 4
- Minimum number of samples required to split an internal node (*min_samples_split*) = 5
- Number of boosting stages to perform (*n_estimators*) = 1000

Both MLP architectures featured 30% dropout at each layer to prevent overfitting, the activation used a ReLU function for the hidden layers, and linear activation for the final output layer.[3] MLP's were trained on batch sizes of 32, using the Adam stochastic gradient descent optimizer.[4] Convergence in MAE associated with the validation data was used to truncate MLP training, using the EarlyStopping method in Keras. This provided maximum training, while avoiding potential overfitting from extensive training beyond numerical convergence.

6. Metrics summary

Table S2 reports a complete comparison of ML model training metrics across each of the methods considered in the present article.

Table S2. Mean absolute errors (MAE) at each ML method and training dataset size.

	**MLP *shallow***	**MLP *deep***	**GBDT**	**LR**
		80% Training data		
**CCF- E_{mix}**	-	0.018	-	0.020
**CCF- E_{gap}**	-	0.089	-	0.100
**CME- E_{mix}**	-	-	-	0.001
**CME- E_{gap}**	0.016	0.008	0.010	0.029
		50% Training data		
**CME- E_{gap}**	0.017	0.010	0.010	0.029
		30% Training data		
**CME- E_{gap}**	0.020	0.011	0.012	0.030
		10% Training data		
**CME- E_{gap}**	0.019	0.016	0.015	0.030

7. Information about codes and data

We provide the following resources in open access repositories:

Data:

- All data is available in a Zenodo repo – with a persistent DOI associated to ensure continuation of availability: https://doi.org/10.5281/zenodo.4736810
- The repo contains both the raw data (in .csv format) and pre-processed data (in .pkl format, ready for training the ML models).
- Distributed under Creative Commons Attribution International 4.0 License.

Code:

- All the code required to reproduce our results is available on a github repo (https://github.com/scott-midgley/Machine-Learning-for-Solid-Solutions).
- The github repo is linked to the Zenodo data repo – by combining the two repositories anyone should be able to reproduce all our results.
- The code repo includes a conda environment specification (in .yml format), so that it is possible to recreate the environment settings that we used to perform the study.
- All code is written as Jupyter notebooks, with full instructions and comments to make it easy to follow.
- The repo contains instructions on how to run the codes and recreate the appropriate environment and link to the data.
- Distributed under MIT License.

Models:

- The models can be recreated from the code and data, typically within a short time.
- The deep MLP networks take longer to train, so we have also provided the pre-trained models as serialized objects in .h5 files, which can be easily loaded and re-run. The code for doing so is also provided in notebooks.

For more detailed information, please refer to the README files within each repository.

References

1. Sanchez, J. M.; Ducastelle, F.; Gratias, D., Generalized Cluster Description of Multicomponent Systems. *Physica A: Statistical Mechanics and its Applications* **1984**, *128*, 334-350.
2. http://Exciting-Code.Org/Carbon-Cell-Tutorial.
3. Agarap, A. F., Deep Learning Using Rectified Linear Units (Relu). *arXiv:1803.08375* (2018).
4. Kingma, D. P.; Ba, J. A., Adam: A Method for Stochastic Optimization. *arXiv:1412.6980v9* (2017)
